# Novel *SPEF2* Variant in a Japanese Patient with Primary Ciliary Dyskinesia: A Case Report and Literature Review

**DOI:** 10.3390/jcm12010317

**Published:** 2022-12-31

**Authors:** Mayako Mori, Takashi Kido, Noriho Sakamoto, Mutsumi Ozasa, Kumiko Kido, Yasuko Noguchi, Takatomo Tokito, Daisuke Okuno, Hirokazu Yura, Atsuko Hara, Hiroshi Ishimoto, Takashi Suematsu, Yasushi Obase, Yoshimasa Tanaka, Koichi Izumikawa, Kazuhiko Takeuchi, Hiroshi Mukae

**Affiliations:** 1 Department of Respiratory Medicine, Graduate School of Biomedical Sciences, Nagasaki University, Nagasaki 852-8501, Japan; 2 Department of Pathology, Graduate School of Biomedical Sciences, Nagasaki University, Nagasaki 852-8520, Japan; 3 J-One, Nagasaki 852-8505, Japan; 4 Central Electron Microscope Laboratory, Graduate School of Biomedical Sciences, Nagasaki University, Nagasaki 852-8523, Japan; 5 Center for Medical Innovation, Nagasaki University, Nagasaki 852-8521, Japan; 6 Department of Infection Control and Education Center, Nagasaki University Hospital, Nagasaki 852-8501, Japan; 7 Department of Otorhinolaryngology-Head and Neck Surgery, Graduate School of Medicine, Mie University, Tsu 514-8507, Japan

**Keywords:** ciliary beat frequency, ciliary beat amplitude, high-speed video microscopy analysis, primary ciliary dyskinesia, *SPEF2*

## Abstract

Primary ciliary dyskinesia (PCD) is a genetic and congenital disease associated with an abnormal ciliary ultrastructure and function and is estimated to affect 1 in 15,000–20,000 individuals. A PCD diagnosis can be achieved by genotyping. Here, we performed whole-exome analysis for the diagnosis of PCD and described the detailed clinical characteristics of the case. A 39-year-old Japanese woman with sinusitis and bronchiectasis without situs inversus had had upper and lower respiratory symptoms since childhood and had received long-term macrolide therapy without an accurate diagnosis. A moderate deterioration of cilia function was observed by high-speed video microscopy analysis; additionally, the number of cells with moving cilia was fewer than that in patients without PCD. Electron microscopy revealed no apparent structural abnormalities. We performed whole-exome analysis and identified novel biallelic variants of *SPEF2* in the homozygous state (c.1860_1861insCT). We confirmed the absence of SPEF2 protein expression in the cilia of the nasal mucosa using fluorescent immunostaining. Accordingly, she was diagnosed as having PCD with the *SPEF2* variant. The present case suggests that the deterioration of cilia function is moderate, the number of respiratory cells with moving cilia might be reduced, and the respiratory condition could be severe in patients with PCD with the *SPEF2* variant.

## 1. Introduction

Primary ciliary dyskinesia (PCD) is a genetic and congenital disease associated with abnormal ciliary function and the overall minimum global prevalence is calculated to be at least 1 in 7554 individuals [1,2,3]. Repeated lower respiratory tract infections due to ciliary dysfunction result in the progressive destruction of the lung, which can lead to severe and fatal pulmonary dysfunction. Diagnosis is difficult, and the majority of patients visit a physician more than 30–50 times before diagnosis [4,5]. All diagnostic tests, including nasal nitric oxide measurements, analyses of ciliary beats, ultrastructural analyses of cilia, and molecular testing for mutations in PCD genes, require specific instruments and specialists, and all the tests have limitations and are not perfect for diagnosis [6]. New PCD causative genes have been discovered each year, and genotyping may identify a genetic cause in 50–75% of cases. In addition, each mutation is associated with distinct clinical characteristics of PCD, such as disease severity, ciliary beat patterns, and ultrastructural abnormalities of cilia [7,8,9]. 

Here, we performed whole-exome analysis for the diagnosis of PCD, identified the novel biallelic variants of *Sperm Flagellar 2* (*SPEF2*) in the homozygous state, c.1860_1861insCT, which has not previously been reported, and described the detailed clinical characteristics of the case.

## 2. Case Presentation

**Figure 1:**
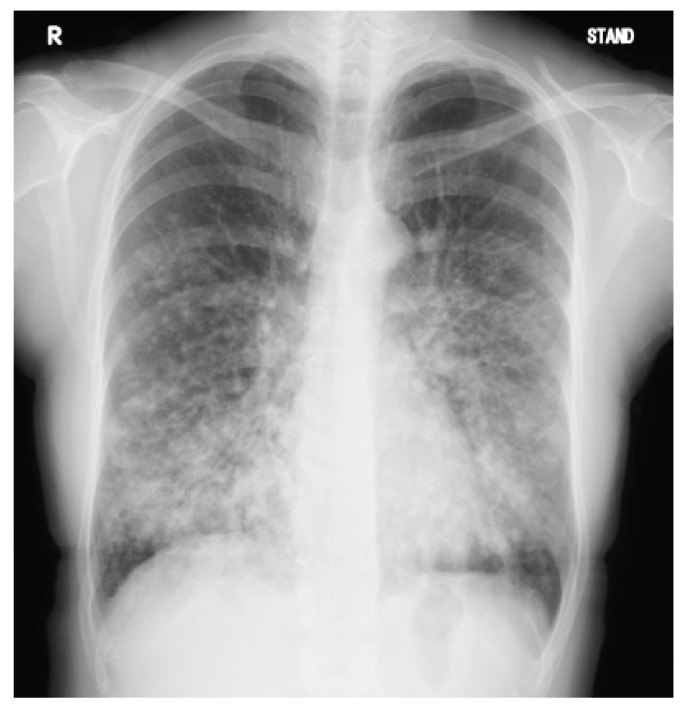
A chest radiograph of the patient at 39 years of age. Diffuse nodules, consolidation, and bronchial wall thickening predominantly in the lower lung fields are observed.

A 39-year-old Japanese woman with sinusitis and bronchiectasis had had upper and lower respiratory symptoms, such as a wet cough, since childhood. At 13 years of age, the patient was diagnosed with sinobronchial syndrome and received long-term macrolide therapy. At the age of 25 years, the patient was referred to our hospital, and a nasal biopsy for the diagnosis of PCD was performed; however, no cilia were found in the tissue, and the result did not reach the level of diagnosis. *Pseudomonas aeruginosa* was detected in the patient’s sputum at the age of 25 years. Repeats of acute bronchiectasis exacerbations and a gradual decline in respiratory function were also shown. At 39 years of age, home oxygen therapy was initiated, and registration on a waiting list for lung transplants from brain-dead donors was applied for. The pulmonary function test results at the age of 39 years were as follows: forced vital capacity, 2.27 L (78.5% of the predicted value); forced expiratory volume in 1 s, 1.18 L (43.4% of the predicted value); and carbon monoxide diffusion capacity of the lung, 16.5 mL/min/mmHg (83.0% of the predicted value). The fraction of exhaled nitric oxide levels was low (5 ppb; the mean levels of Japanese individuals are approximately 15 ppb). Chest radiography (Figure 1) revealed diffuse nodules, consolidation, and bronchial wall thickening, predominantly in the lower lung fields. 

**Figure 2:**
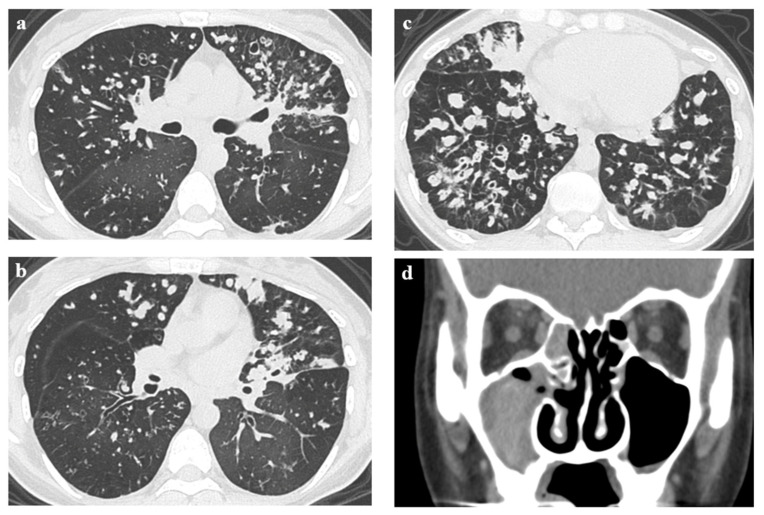
(**a**–**c**) Representative images of CT scan. Chest CT scan shows diffuse bronchial wall thickening, bronchial dilatation, and mucus plugs with bilateral middle and lower lung fields predominant; (**d**) A sinus CT scan shows fluid retention and mucosal thickening occupying the right maxillary sinus.

Chest high-resolution computed tomography (HRCT) revealed diffuse bronchial wall thickening, bronchial dilatation, and mucus plugs with predominant bilateral middle and lower lung fields (Figure 2a–c). A sinus computed tomography (CT) scan showed fluid retention and mucosal thickening occupying the right maxillary sinus, suggesting chronic sinusitis (Figure 2d). 

**Figure 3:**
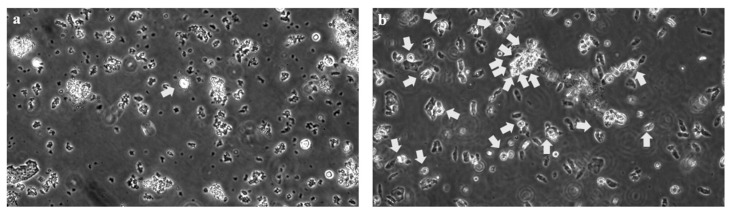
Captured picture (**a**,**b**) of videos (Video S1a,b) during the assessment of the ciliary beat. Ciliary beats for the present case (**a** and Video S1a) and healthy participants (**b** and Video S1b) were assessed using cells obtained by nasal mucosal brushing. Videos were recorded with a high-speed video camera (HAS-L1, DITECT). Compared with healthy participants, the velocity amplitude, angle, and coordination of ciliary beats of the patient were slower, lower, and poorer. Although only one cell in the center of the picture (white arrow) can be observed in the present movie of this case (**a** and Video S1a), many cells with moving cilia were observed in healthy participants (**b** and Video S1b). The original magnification of the optical microscope is 400.

The ciliary beat frequency (CBF), ciliary beat amplitude (CBA) (assessed by protractor, Figure S1), and ciliary coordination were immediately (within 10–30 min) assessed using cells obtained by nasal mucosal brushing with a high-speed video camera (HAS-L1, DITECT, Tokyo, Japan). One cell in five movies, five cells in total, of each right and left nostril was assessed in the medium (1:1 bronchial epithelial cell growth medium and Dulbecco’s modified eagle medium) [10]. Videos attached to a microscope were recorded at high speeds (more than 100 fps) and were replayed more slowly (5–30 fps) for evaluation using software (HAS-X viewer Ver.1.3.5.0., DITECT). The right median CBF and CBA were 4.63 Hz and 80 degrees, respectively, and the left median CBF and CBA were 2.75 Hz and 50 degrees, respectively, suggesting moderate deterioration of cilia function at the levels of our laboratory data. In patients without PCD, the median CBF and CBA are approximately 10 Hz and 100–150 degrees, respectively, in our laboratory data. In addition, ciliary coordination was moderately poorer than that of healthy participants and better than that of a patient with outer dynein arm defects in our laboratory data. Although five cells in the right and left nostrils were assessed, fewer cells with moving cilia were observed than in patients without PCD (Figure 3, Video S1). 

**Figure 4:**
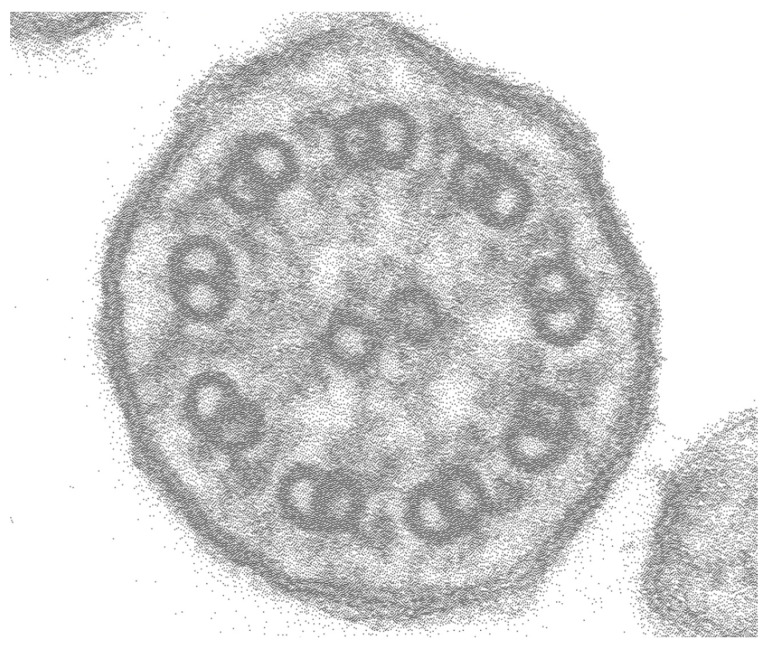
Representative image of electron microscopy. There are no obvious structural abnormalities in nasal cilia.

**Figure 5:**
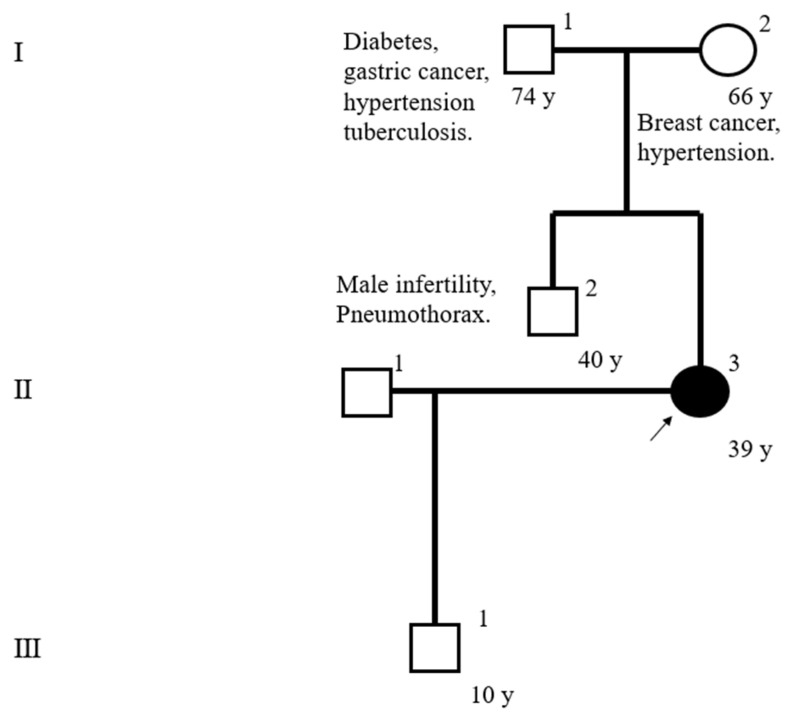
The patient’s pedigree. The patient’s brother was diagnosed male infertility.

Electron microscopy did not reveal any obvious structural abnormalities in cilia (Figure 4), and genetic testing showed no variants in cystic fibrosis-related genes. The pedigree is shown in Figure 5. 

**Figure 6:**
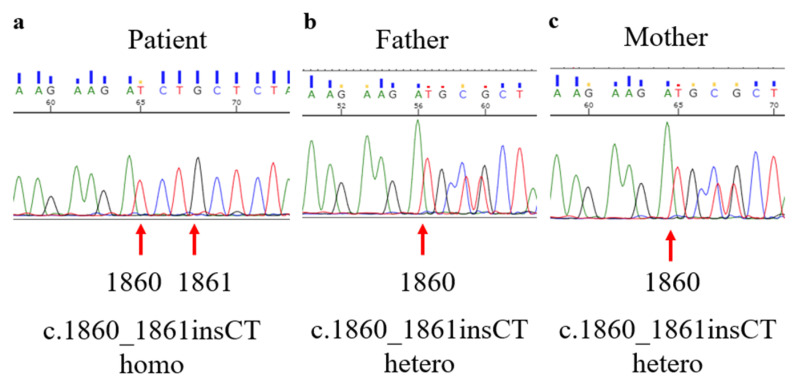
Genetic analysis using Sanger sequencing of (**a**) the patient, (**b**) the patient’s father, and (**c**) the patient’s mother. Stop mutation c.1860_1861insCT (p.Ala621LeufsTer59) in *SPEF2* in a homozygous state was observed in (**a**) the patient and the heterozygous mutations were found in (**b**,**c**) the patient’s father and mother. *SPEF2 = sperm flagellar 2.* Green: adenine (A). Black: guanine (G). Red: thymine (T). Blue: cytosine (C).

We performed a PCD gene panel, whole-exome sequencing, and Sanger sequencing as described previously [11], and identified the frameshift mutation c.1860_1861insCT (p.Ala621LeufsTer59) in *SPEF2* in a homozygous state (Figure 6). 

**Figure 7:**
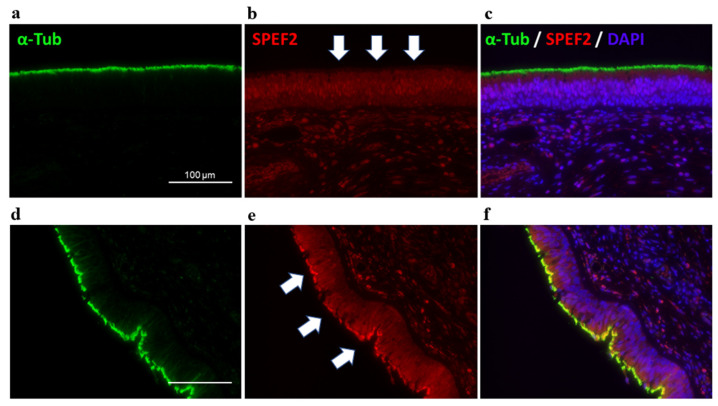
Immunofluorescence analysis. (**a**–**c**) Images of sinus tissues obtained by surgery for chronic sinusitis of the present case; (**d**–**f**) the other case of PCD used as a positive control. Anti-SPEF2 (HPA039606, Atlas antibodies, Bromma, Sweden) (red) is stained with anti-acetylated tubulin (T7451, Sigma-Aldrich, St Louis, MO, USA) (green) to visualize the entire ciliary axonemes, and with 4′,6-diamidino-2-phenylindole (17507, AAT Bioquest, Pleasanton, MO, USA) (blue) to show the nucleus as well. The white arrows and yellow color, indicate the presence of SPEF2 in the cilia in the image of the control (**e**,**f**). In (**b**,**c**), a lack of SPEF2 expression can be confirmed. Scale bar: 100 μm.

The parents had heterozygous variants, suggesting a compound heterozygous inheritance trait. We also confirmed the absence of SPEF2 protein expression by fluorescent immunostaining in the cilia of the sinus tissues that were obtained by surgery for chronic sinusitis (Figure 7) [12]. Accordingly, she was diagnosed as having PCD with the *SPEF2* variant.

## 3. Discussion

Herein, we present an *SPEF2* variant in a 39-year-old Japanese woman with progressive respiratory dysfunction. Currently, only a 31-year-old woman from Germany and six men (ages unknown) with infertility from China were reported in 2020 as cases of PCD with the *SPEF2* variant [12,13]. In addition, some *SPEF2* variants in patients with male infertility have been reported in China [14]. In the present case, we identified novel variants of *SPEF2* in the homozygous state (c.1860_1861insCT), which have not been reported previously. In patients with PCD, homozygous variants of c.1639C>T from Germany as well as homozygous variants of c.2507 + 5delG, c.2649dupA, and c.C4096T and heterozygous variants of c.3400delA and c.3922dupA from China have been shown. 

*SPEF2* is widely expressed in cilia-related organs, such as the lungs, spleen, trachea, brain, and testis [15,16]. *SPEF2* is a component of the central pair complex (CPC) of cilia, and *SPEF2* mutations cause symptoms associated with ciliary dysfunction, such as male infertility and/or PCD [12,13,14]. In ultrastructural analyses using transmission electron microscopy, morphological abnormalities of the sperm flagellum have been observed in patients with *SPEF2* mutations, but no visible ultrastructural abnormalities of the respiratory cilia have been observed [12,13]. Thus, we confirmed the diagnosis via the lack of *SPEF2* by fluorescent immunostaining, in addition to gene analyses, similar to the cases from Germany and China. 

Although little is known regarding the ciliary beat motion of patients with PCD with *SPEF2* variants, the present case showed the moderate deterioration of the CBF (4.63 and 2.75 Hz) and CBA (80 and 50 degrees) (median, right and left nostrils, respectively), and moderately poorer ciliary coordination. Only one case from Germany showed the deterioration of the CBF (5 to 2.5 Hz) without information on the CBA and ciliary coordination [12]. Ciliary function is closely associated with specific ultrastructural defects, and the CBF of minor or no obvious ultrastructural abnormalities is relatively mildly reduced compared with the CBF of “classic” PCD with outer dynein arm defects or no dynein arms [1,17]. As *SPEF2* is a component of the CPC and is not related to the dynein arms of cilia, the moderate deterioration of the CBF in patients with *SPEF2* variants is reasonable. The CBA and ciliary coordination were also moderately poor in the present case. Furthermore, fewer cells with moving cilia were observed in the present case than in healthy participants. It was also shown that the total number of cilia was greatly reduced, and the apoptotic signal from the nasopharynx increased in epithelial cells, which might be caused by chronic inflammation or partly attributed to the ciliogenesis defect triggered by *SPEF2* deficiency [13]. We believe that these results are reasonable and very important, but the evidence is very limited, and the accumulation of knowledge is expected. To avoid the effects of secondary damage by sampling and infection, it is recommended to confirm with repeated evaluation and/or cell culture samples [8]. Though we evaluated twice (right and left nasal brushing), it might be better to evaluate with further samples, such as bronchial cilia and/or cell culture samples. 

The present case and a case from Germany were reported in female patients, and six cases from China were reported in male patients with infertility [12,13]. All eight patients had upper and lower airway symptoms without a situs inversus. The situs inversus is not observed in patients with PCD with CPC-related gene variants, including *SPEF2* [12]. The predicted value of the forced expiratory volume in 1 s (%FEV_1_) was only 43.4% at 39 years of age and required home oxygen therapy and registration for lung transplant. Furthermore, %FEV_1_ was 50% in a 31-year-old woman from Germany. These two cases from Japan and Germany suggest that reduced lung function in patients with PCD with *SPEF2* variants was relatively severe, although the deterioration of the CBF in these two cases was relatively moderate. Persistent *Pseudomonas aeruginosa* infection, known as a poor prognosis factor, seems to be one of the causes of progression in the present case [1,18,19]. The association of severity, prognosis, and persistent *Pseudomonas aeruginosa* infection in patients with PCD with *SPEF2* variants remains unclear, and an accumulation of evidence is expected.

In conclusion, we present a rare case of PCD with the *SPEF2* variant as a third report, preceded by reports from Germany and China, although the variant (c.1860_1861insCT) has never been reported. The present case suggests that the deterioration of cilia function is moderate, the number of respiratory cells with moving cilia is highly reduced, and the respiratory condition could be severe in patients with PCD with *SPEF2* variants. The number of reported cases is limited, and the accumulation of evidence is expected.

## Data Availability

The datasets for the current case are available from the corresponding author upon reasonable request.

## Supplementary Materials

The following supporting information can be downloaded at: https://www.mdpi.com/article/10.3390/jcm12010317/s1, Figure S1: An illustrative diagram of measuring CBA. Video S1a: Video of cilia of the patient. Video S1b: Video of cilia of healthy participants.

## Abbreviations

CBA	ciliary beat amplitude
CBF	ciliary beat frequency
CPC	central pair complex
PCD	primary ciliary dyskinesia
HRCT	high-resolution computed tomography
%FEV_1_	predicted value of forced expiratory volume in 1 s
