# Supplementary figures and images for: Novel SPEF2 Variant in a Japanese Patient with Primary Ciliary Dyskinesia: A Case Report and Literature Review
Source: J Clin Med. 2022 Dec 31;12(1):317. doi: 10.3390/jcm12010317 (PMC9821625; doi:10.3390/jcm12010317)

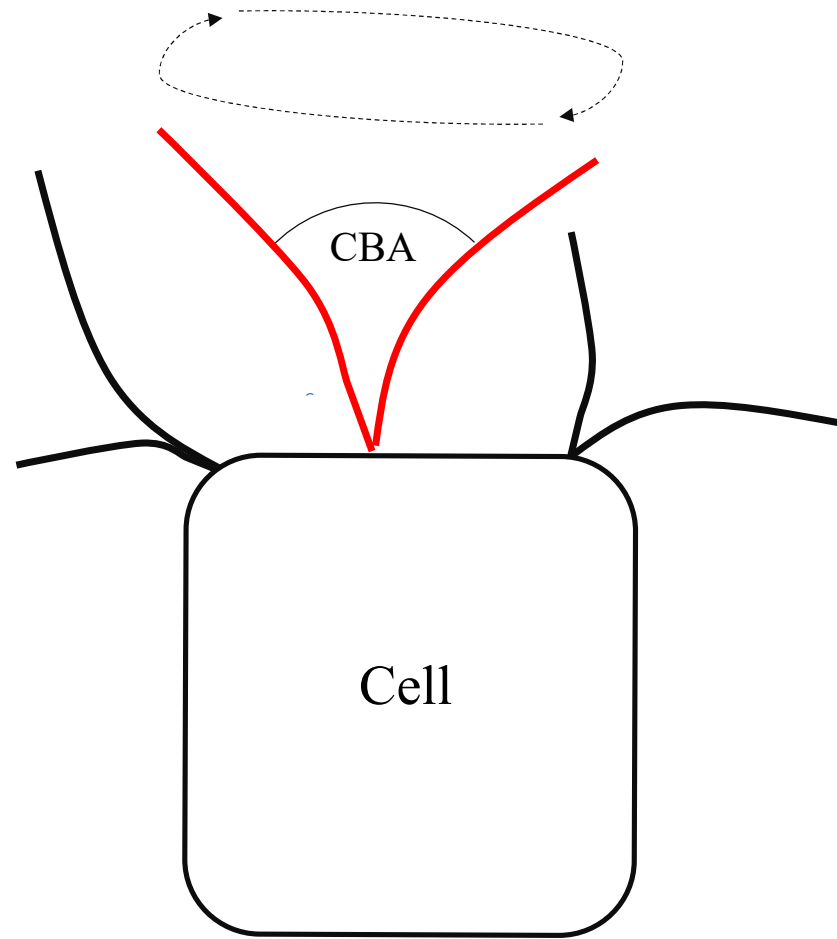

Figure S1: Image of measuring CBA.

Supplement: Supplementary file 1 [file jcm-12-00317-s001.zip › jcm-2086764-supplementary.pdf]
